# Relationship of isotopic variations with spring density in the structurally controlled springs and related geosystem services in Alaknanda Valley, Garhwal Himalaya, India

**DOI:** 10.1038/s41598-022-11762-z

**Published:** 2022-05-10

**Authors:** Aakash Mohan Rawat, Dhirendra Singh Bagri, Sudhir Kumar, Ruchi Badola, Syed Ainul Hussain

**Affiliations:** 1 Department of Geology, Hemvati Nandan Bahuguna Garhwal University, Srinagar Garhwal, Uttarakhand India; 2 National Institute of Hydrology, Roorkee, Uttarakhand India; 3 Wildlife Institute of India, Chandrabani, Dehradun, Uttarakhand India

**Keywords:** Environmental sciences, Environmental social sciences, Hydrology

## Abstract

As a traditional water source, springs are vital for Himalayan communities and it is essential to consciously focus on springs conservation. We report oxygen isotopes (δ^18^O) of spring water before, within, and after the tectonically active zones of the Alaknanda Valley, Uttarakhand. Higher variation of δ^18^O in the spring waters is found in highly tectonically disturbed zone i.e., Zone-2 with δ^18^O range − 4.9‰ to − 9.0‰ compared to tectonically less disturbed zones: Zone-1 and Zone-3 with δ^18^O value range − 7.9‰ to − 9.9‰ and − 7.4 to − 10.2‰ respectively. We hypothesize that the highly active thrust zones (Zone-2) with increased permeability compared to other Zones, manifested as greater spring density, results in higher water recharge in Zone-2. Very high to high spring density stretches are dominant in Zone-2 compared to Zone-1 and Zone-3. Stretches in Zone-2 with high spring density formed due to its highly tectonically active nature leads to the higher isotopic variation in Zone-2. The study also identifies the geosystem services provided by thrust zones as water resources to the local people and need of conservation modalities to manage the spring water resources in the thrust zones.

## Introduction

Many accounts associated with the earthquake-induced permeability enhancements and subsequent release of the new water sources have been documented worldwide^1–3^. Further, the coseismic hydrological changes propose the groundwater mixing among different aquifers through new cracks^4^, which is the manifestation of the enhanced permeability due to the earthquakes. Stable isotopes of water have been used as proxy^5^ to show that that earthquake enhanced permeability and release water from mountains as a subsurface hydrogeological response to the 2016 Mw 7.0 Kumamoto earthquake. The stable isotopic variations have been used as a direct fingerprinting tool to examine the changes in between before and after earthquakes^2,3,6^. On the other hand, the variation in the isotopic ratios (^18^O/^16^O) of oxygen has generally been attributed to evaporation of meteoric water^7^, together with ^18^O-shift by the water–rock interaction^8^ and changes of the oxygen isotopes before earthquakes^9–11^. Also, the isotopic variation with altitude has been worked out by^12,13^. This progressive depletion of the heavier isotope in rain with altitude, as the cloud ascends, is often referred to as the “altitude effect”. Thus, most of the studies are focused either on the earthquake or altitude-regulated isotopic variations (altitude effect) or due to the other fractionation processes. Other factors like the very high abundance of springs sources or very high spring density in some stretches and the presence of active thrust zones may also also bring changes in the stable water isotopes giving the indication of water recharge in the zone. As the stable isotopes of oxygen indicate the recharge sources of the water^14–16^, the present work has been carried out to understand the impact of tectonics on isotopic composition of spring water.

In the present work, we tried to decipher the first account of the thrust-controlled stable oxygen isotope variation in the spring waters of Alaknanda valley located in Garhwal Himalayas, Uttarakhand, India.

## Study area

**Figure 1:**
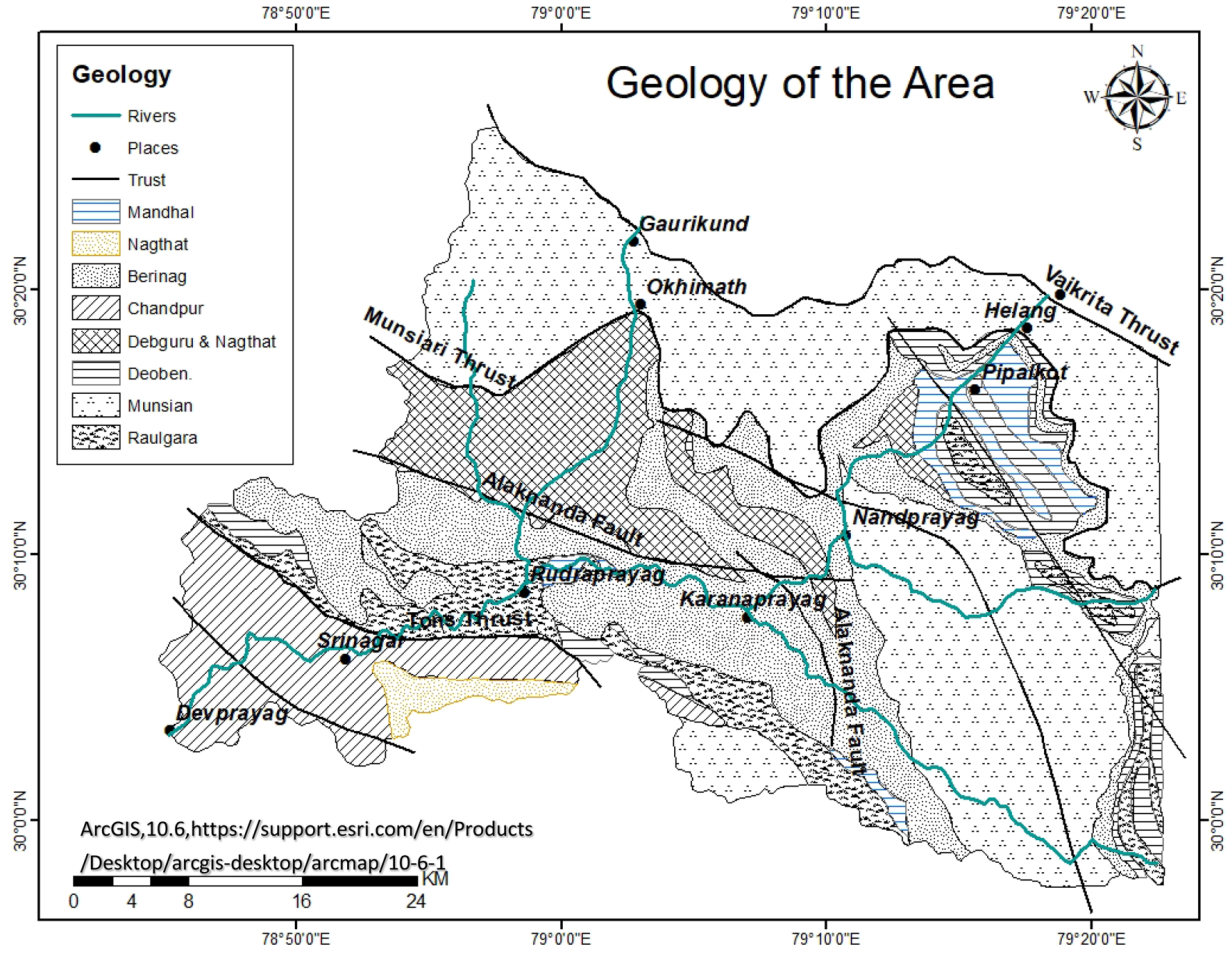
Geology of area (modified after^41^).

The study area is located in the Alaknanda Valley of the Garhwal Himalayas, the sub-tropical, humid, and temperate type of climate zone encompassing the coordinates of 30°0’N–31°0’N latitude and 78°45’E–80°15’E longitude as shown in Fig. 1. The river basin covers an area of 11,085 km^2^. The study area is characterized by elevated, rugged mountain landscapes, V-shaped valleys, river terraces, flood plains, etc.

### Geological and structural setting

**Figure 2:**
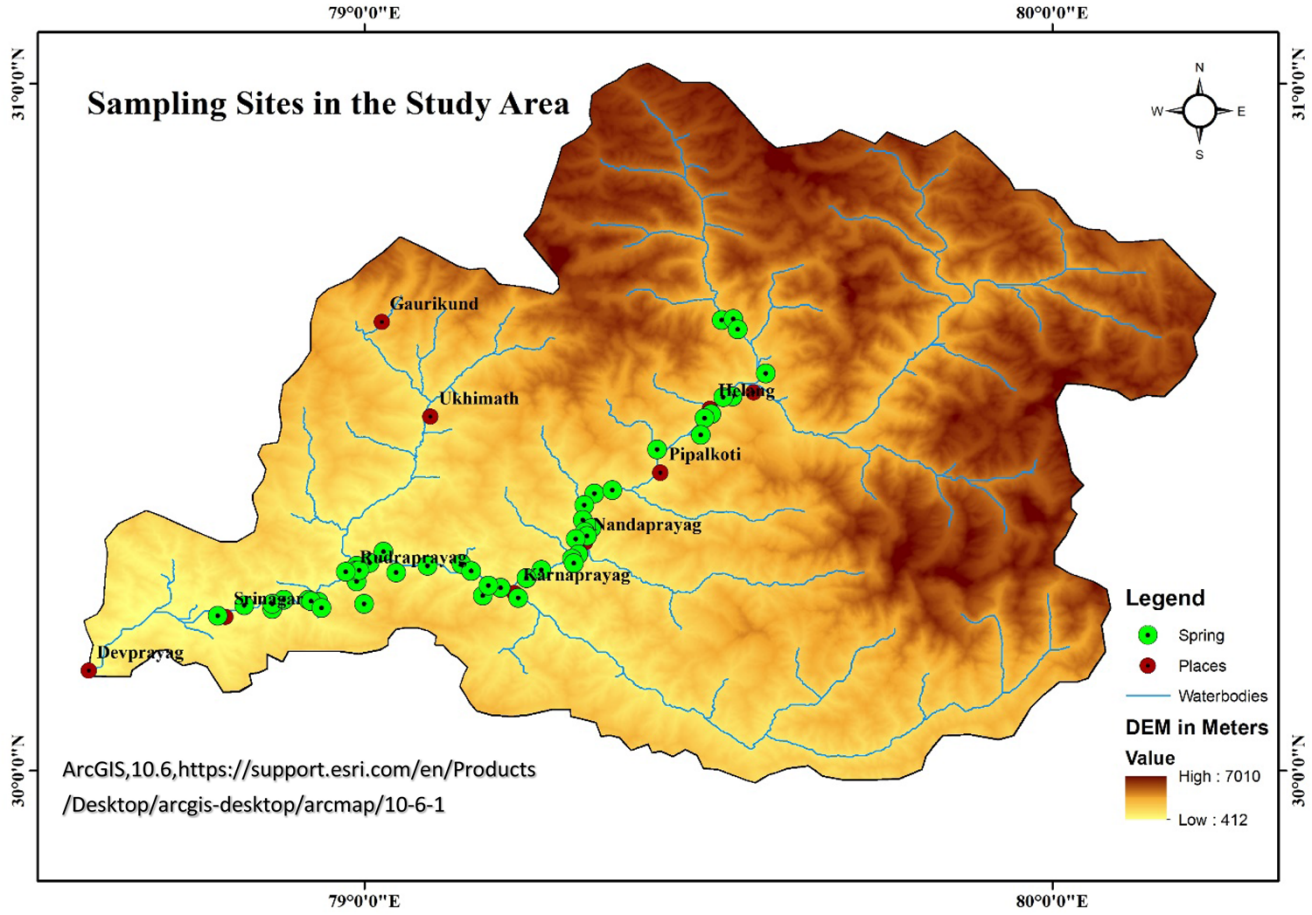
Sampling sites within the study area georeferenced and modified after Shukla et al.^17^.

The Alaknanda river basin lies between the sediments of Tehthyan Himalayan sediments and rocks of Lesser Himalayan sequence. The basin has its southern portion underlain by limestone, phyllite, schists and quartzites of the Lesser Himalayan series ^24–27^. The middle part is dominated by the gneisses of Higher Himalayan series and the northern part is underlain by meta-sedimentary units of the Tethyan series. The High-grade metamorphic rocks (gneisses, metabasics, quartzites, high-grade schists, and granites), with limited carbonate and calc-silicate rocks, are a part of the Higher Himalayan Crystalline^27–29^, and these crystalline are termed as Vaikrita Group ( where the upper crystalline portion lies between South Tibetan detachment system with the acronym (STDS) and Main Central Thrust with the acronym (MCT)-I or Vaikrita Thrust), Jutogh or Munsiari Group (middle crystalline areas between MCT-I and MCT-II or Munsiari thrust) and Chail or Ramgarh Group (lower crystalline zone between MCT-II and MCT-III or Ramgarh Thrust)^26,30–32^ Figs. 1 and 2. Below the Vaikrita Thrust, the lower unit of the Himalayan crystalline core predominantly constitutes lower-amphibolite facies, schists and low-grade gneisses related to Lesser Himalayan crystalline sequence (LHCS). In the Alaknanda valley from Helang (south of Joshimath) to Badrinath these rocks are well exposed. These are certain portions that show the presence of carbonaceous shales, limestones and dolomites of the Ramgarh Formation; quartz arenites and mafic volcanic rocks belonging to Berinag Formation; and phyllites and siltstones lying in the Chandpur Formation^31–35^ (Figs. 1 and 2). The Lesser Himalaya zone is bounded by MCT in the north and MBT in the south, is vivisected by the North Almora Thrust (NAT) and South Almora Thrust (SAT) to the north and south of the Dudatoli syncline, respectively^26,27^.

With the help of morpho-tectonic active map, the area has been divided into three zones such that the Zone-1 and Zone-2 lie in tectonically highly and very highly active zones respectively, while Zone-3 lies in moderately active zone^17^. Also, the earthquake density is very high in Zone-2, moderate in Zone-1, and low in Zone-3^17^.

## Methodology

### Data collection, measurement and analysis

Field work was conducted in the month of March, 2017. Springs water samples (*n* = 96) were collected in pre-cleaned high-density polyethylene bottles (HDPE) (Supplementary information Table.1). The bottles were filled without air bubbles and tightly capped to avoid any evaporation and exchange with the atmospheric moisture. The elevation, time and location were recorded for individual sample. The isotopic analyses of water samples were carried out at Isotope Hydrology Laboratory, National Institute of Hydrology, Roorkee, India. The collected samples were analysed for stable isotopic ratio of oxygen (δ^18^O) using Dual Inlet Isotope Ratio Mass Spectrometer following CO_2_ equilibration methods with systematically described steps and standards in the measurement^36,37^.

**Figure 3:**
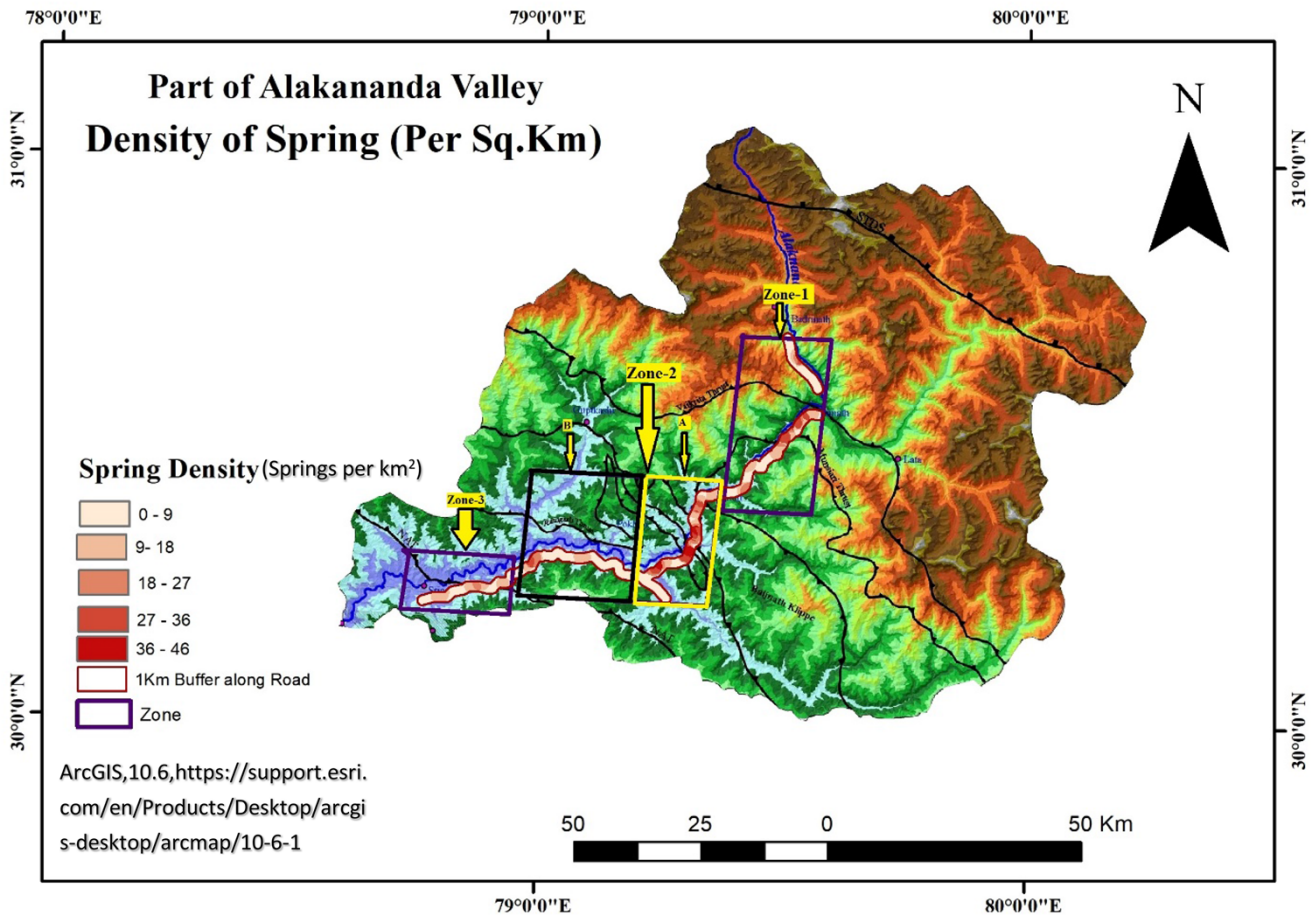
Spring density in per km^2^ in Zones-1, 2 and 3 and in compartmentalised Zone-2 (i.e., **A** and **B**), georeferenced and modified after^17^).

**Figure 4:**
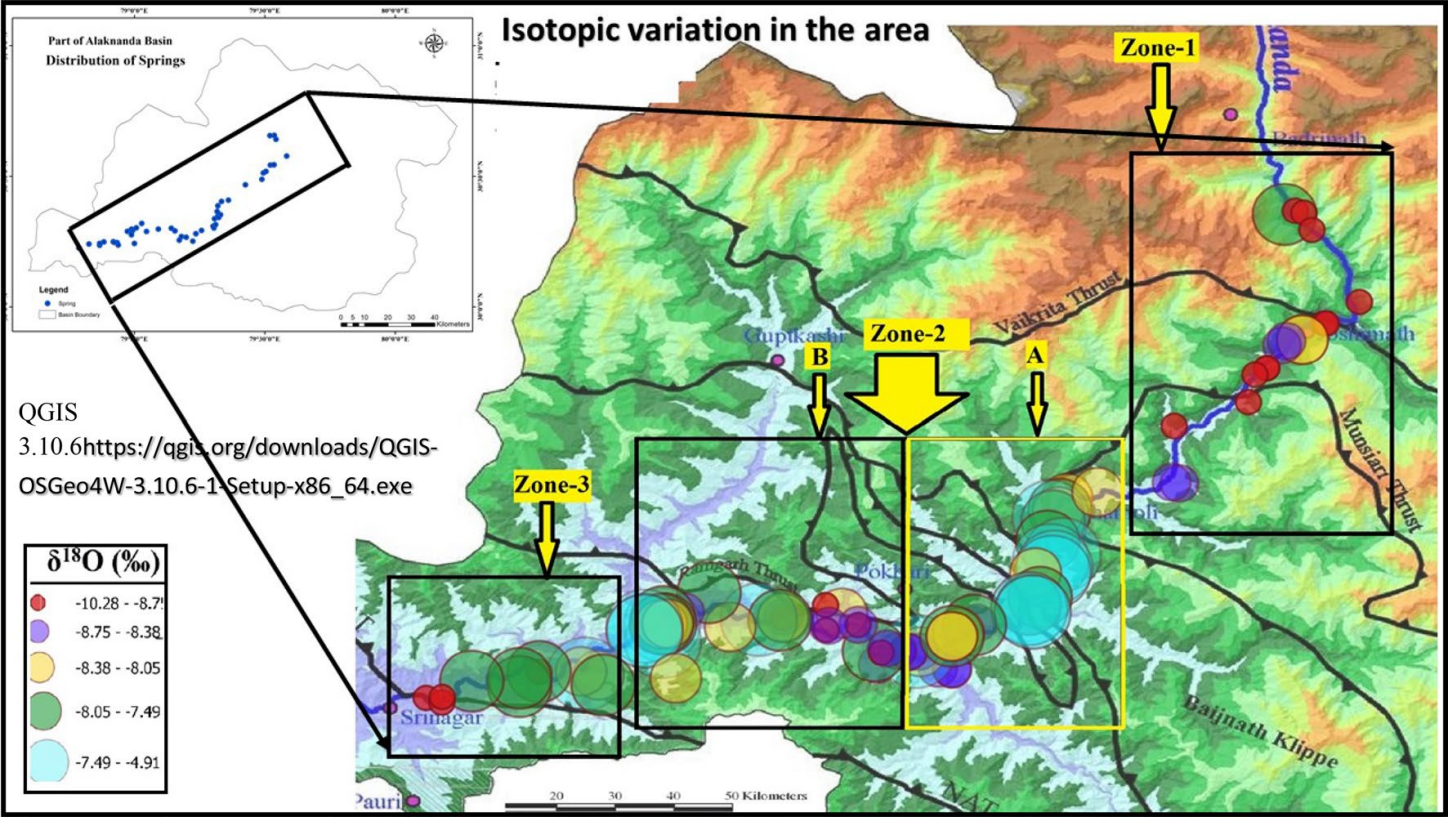
δ^18^O (‰) variation/diversity within the zones (1, 2, 3) and in compartmentalised Zone-2 (i.e., **A** and **B**) georeferenced and modified after (Shukla et al.^17^). **A** and **B** in Zone-2 accounts for maximum species of δ^18^O (‰) compared to Zone-1 and Zone-3.

Figure 1 showing the geology of the area have been derived by georefrencing and modifying the Fig. 2 of ^41^ Rana et al. by using ArcGIS Desktop 10.6 software (https://support.esri.com/en/Products/Desktop/arcgisdesktop/arcmap/10-6-1). The DEM map for the area is shown in Figs. 2 and 4 are created by georeferencing Fig. 1 of Shukla et al.^17^ and superimposed with spring data using Arc GIS Desktop10.6 software and QGIS 3.10.6 software (QGIS 3.10.6 https://qgis.org/downloads/QGIS-OSGeo4W-3.10.6-1-Setup-x86_64.exe) respectively. The spring density shown in Fig. 3 is made by georeferenceing Fig. 1 of Shukla et al.^17^, using ArcGIS Desktop10.6 software. According to the criteria of^17^, the area with spring data is divided into three zones i.e., Zone-1, Zone-2 and Zone-3, as shown in Figs. 3 and 4, where the Zone-2 is “very highly tectonically active” zone, Zone-1 is “highly tectonically active” zone and Zone-3 is “moderately tectonically active” zone. Data analysis to evaluate the δ^18^O variation in boxplots was done through R studio 1.4.1717 software (https://educe-ubc.github.io/r_and_rstudio.html). The three zones viz. Zone-1, Zone-2, Zone-3 were mapped. The Zones 1, 2 and 3 are Lambagad-Joshimath-Helang zone, Chamoli-Karanprayag-Rudraprayag zone and Srinagar zone respectively. In order to establish a balanced comparison with dataset in three zones and to support the isotopic variation due to tectonics the spring density have been calculated using the point density tool in ArcGIS software as shown in Fig. 3. The spring density was calculated by finding the number of springs in 1 km^2^ within a buffer of 1 km along the road in study area and is measured in number of springs per km^2^. To minimise the contribution of altitude effect the Zone-2 is further divided into two smaller sub-zones i.e., **A** (outlined yellow, with high density stretches) and **B** (outlined black, with low density stretches) and thus a balanced dataset for each zone was established. Also the high to very high spring density stretch in Zone-2 i.e., **A**, is compared with the similar type of density stretch of Zone-1 and low spring density stretch of Zone-2 i.e., **B**, is compared the same density stretch of Zone-3 for a balanced comparison. The spring density is used to avoid the comparison biasness that could occur due to bigger size of Zone-2 than Zone-1 and Zone-3. To better explain the isotopic variation of δ^18^O the concept of δ^18^O diversity is introduced in zones under study. The diversity here means the various different species of δ^18^O mentioned in legend that a zone holds and is explained in Fig. 3.

## Results

### Spring density

As shown in Fig. 3, Zone-1 is dominated by high spring density stretches (27–36) to medium spring density stretches (18–27) in the vicinity of Vaikrita and Munsiari Thrusts, and low (9–18) to very low (0–9) springs density stretches away from said thrusts. The compartment A of Zone-2 is dominated by very high (36–45) to high (27–36) to medium (18–27) spring density stretches in the vicinity of Munsiari and Ramgarh Thrusts and low (9–18) to very low (0–9) spring density stretches, away from the said thrust. The compartment B of Zone-2 is dominated by low (9–18) to very low (0–9) spring density stretches amid low vicinity of Ramgarh Thrust, and high (27–36) to medium (18–27) spring density stretches in minority as it approaches to NAT. Zone-3 accounts for the medium (18–27) to low (9–18) spring density stretches in the vicinity of NAT (North Almora Thrust) and very low (0–9) spring density stretches away from NAT (Supplementary Information).

### Isotopic characteristics of spring water

**Figure 5:**
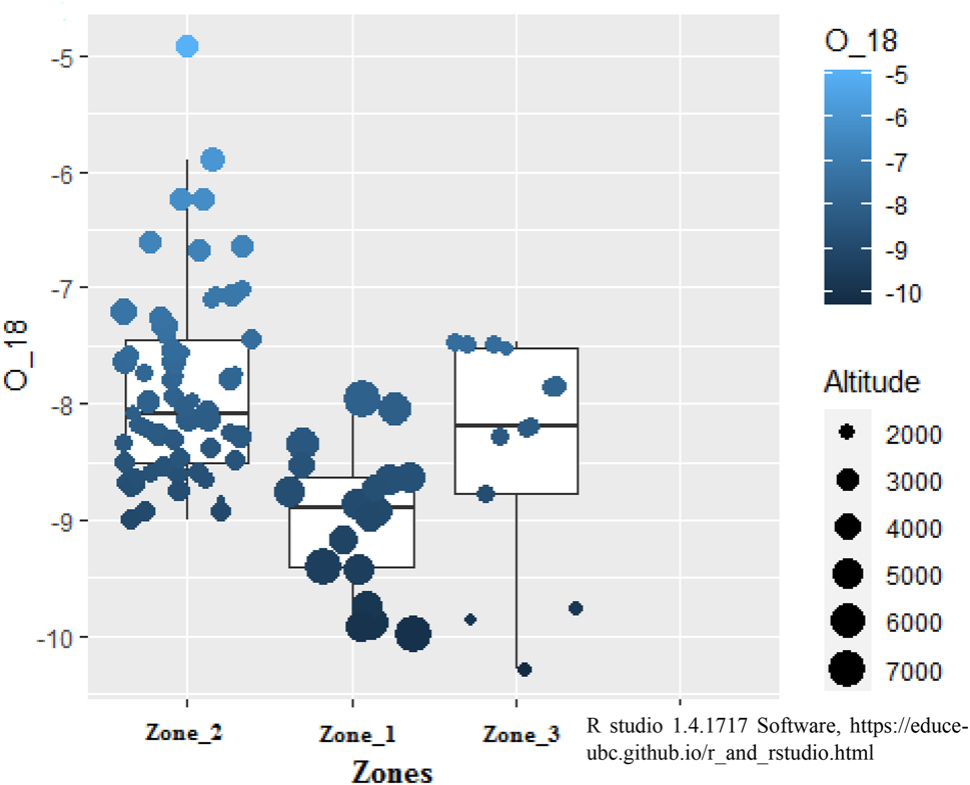
δ^18^O (‰) variation within the zones (1, 2, 3). The altitude variation is quite visible among different zones but is minimised within the particular zone. Response to reviewer 1. Comment for Figure: The third zone has error bar but is too short as it adjusts according to the data. Hence no changes have been made.

The isotopic composition of (δ^18^O) spring water in the area reflects a wide variation within the three zones. It varies from − 7.9 to − 9.9‰ (range: 2.0 ‰) in Zone-1. The variation in Zone-2 is from − 4.9 to − 9.0‰ with the range of 4.0 ‰. The Zone-3 i.e., Srinagar zone follows the variation of − 7.4‰ to − 10.2 ‰ showing the range of 2.8‰. Figures 4 and 5 show the geographical distribution of stable oxygen isotope (δ^18^O) variation in the designated zones. The Zone-2 which is compartmentalised into **A** and **B** as shown in Fig. 4, shows high diversity of δ^18^O species. The **A** compartment of Zone-2 shown in Fig. 4 shows the diversity of δ^18^O species from turquoise blue -green-yellow-violet compared to Zone-1 with diversity of δ^18^O species from and red-blue-yellow-green. Also the turquoise blue in δ^18^O legend suggests maximum variation (− 7.49 to − 4.91) as shown in legend in Fig. 4 after the balanced sampling. The **B** compartment of Zone-2 shown in Fig. 4 shows the diversity/variation from sky blue-green-yellow- turquoise blue-red compared to Zone-3 with diversity/variation from red-green- turquoise blue of Fig. 4 suggesting higher variation in **B** compared to Zone-3.The shape-wise mapping of stable isotope values (δ^18^O) in Fig. 4 shows the full range of isotope (δ^18^O) values randomly distributed indicating higher variation compared to Zone-1 and Zone-3. Thus the high variation within the compartmentalised zones, **A** and **B** suggests the variation may be due to highly tectonically disturbed zone also, rather than just due to the altitude effect which has been minimised by the compartmentalised zones in Zone-2. Altitudinal mapping in the Box plot as in the Fig. 5 shows a low spread of oxygen isotope in low and high altitude zones (Zone-3 and Zone-1 respectively) and a higher spread in Zone-2 that lies between Zone-1 and Zone-3. The corresponding ranges in Zones-1, 2 and 3 are 2.03, 4.09 and 2.82 respectively. The aforesaid range in the zones suggests the stable oxygen isotope (δ^18^O) anomalies as one moves to Zone-2 from Zone-1 and Zone-3.

It is clearly visible from Fig. 5 that the altitude effect becomes prominent as one moves from one zone to another but it is minimised within a given zone. Hence it can be said that large δ^18^O (‰) variation within the Zone-2 compared to others can also be attributed to the spring density rather than only the altitude effect.

## Discussion

The study is a first account that introduces the anomalies of spring density, δ^18^O variation in “tectonically very high”^17^, “high”^17^, and “moderately high”^17^ active zones. The anomalies are expressed in terms of very high (36–45) to high (27–36) spring densities in Zone-2 compared to Zone-1 and Zone-3, which is attributed to the “very highly tectonically active”^17^ stretches in compartment A of Zone-2 in the vicinity of Ramgarh and Munsiari Thrusts as shown in (Fig. 3), giving rise to greater spring densities and high δ^18^O variation in spring water in Zone-2. The A compartment of Zone-2 with very high (36–45), high (27–36) and medium (18–27) spring density stretches is compared to the spring densities of Zone-1 which according to Shukla et al. (2014) lies in the “highly tectonically active zone” is dominated by high spring density stretches (27–36.) to medium spring density stretches (18–27). Thus the higher spring density in compartment A of Zone-2 compared to Zone-1 is attributed to the higher dominance of Ramgarh and Munsyari thrusts in **A** of Zone-2 due to its “very highly tectonically active” nature and lesser dominance of Vaikrita thrust, Munsiari thrust in Zone-1 due to its comparatively tectonically less active nature than Zone-2 as per Shukla et al.^17^ shown in Fig. 3.This gives rise to higher δ^18^O variation in compartment **A** of Zone-2 compared to the Zone-1. Similarly dominance of low to medium spring density in the compartment **B** of Zone-2 is due to the lesser dominance of Ramgarh thrust but the spring density increases to medium and high as one approaches to North Almora Thrust (NAT) and so the variation in δ^18^O which is indicated by the presence of highest variation legend of turquoise blue shown in Fig. 4. Similarly, Zone-3 is dominated by low (9–18) to very low (0 -9) spring density stretches and medium (18–27) spring densities stretches in minority. The spring density in the Zone-3 decreases to very low as one moves away from NAT compared to medium spring density in the vicinity of NAT and hence leads to higher δ^18^O variation in the B compartment of Zone-2 compared to Zone-3. Thus the “highly tectonically active” nature of Zone-2 Shukla et al. dominated by Munsyari (MCT-II) and Ramgarh thrust (MCT-III) compared to less active Zone-1 and Zone-3 containing Vaikrita thrust, Munsiari thrust and NAT respectively Shukla et al.^17^ increases the spring density in Zone-2 compared to Zone-1 and Zone-3 shown in Fig. 3, and this gives an indication of higher water recharge in the Zone-2 which is manifested as the higher δ^18^O anomaly in Zone-2 than in Zone-1 and Zone-3 shown in Figs. 4 and 5. It is thus hypothesized that the dominance of thrust zones in Zone-2 might have enhanced the permeability and may have provided the pathways to enhance flow from mountain aquifers to downslope springs. Certainly, the high and focussed precipitation in Zone-2A^17,41^ could attribute to the high stable isotopic variations in the spring water , but at the same time it is also a zone of active convergence^17,41^, and thus the overlap of the zone of active convergence (highly tectonically active nature of Zone-2A) and focussed precipitation suggests δ^18^O anomaly are not just simply a reflection of rainfall variability only but could also attribute to the permeability enhancement by the zone of active convergence providing additional water recharge to the springs. Shivanna et al.^42^, using δ^18^O showed that the spring water reflects the contributions from both precipitation and groundwater flowing through the weathered and fractured zones further validates the influence of tectonics in providing the additional water to the springs. Further, the spring density and δ^18^O anomaly also increases as one approaches near the vicinity of thrust zones in Zone-1 and Zone-3 despite being the zones of low precipitation^17^, justifying the precipitation alone do not govern the δ^18^O anomaly in the study area. The hypothesis is consistent with the work of^5^ suggesting that fracture systems as the dominant pathways for new waters from mountain aquifers manifested as the high spring density zones in Zone-2**.** Also, higher isotopic variation in spring water of the Zone-2 compared to the Zone-1 and 3 is further supported by the fact that the foot spring waters have more depleted isotopic composition implying a contribution from higher elevations^5^ and thus suggesting additional recharge zones and sources compared to the zones that are less tectonically active. Thus the stable water isotopic variation that we propose is equivalent to the change in isotopic compositions of the spring water, such that the variation in the isotopic composition of spring water gives the indication of the source diversity^2,5^ that recharges the springs but controlled by the highly tectonically active thrust zones in Zone-2 compared to Zone-1 and Zone-3. Consequently, the higher isotopic variation is shown as a farrago of colour mapping of stable isotopes in (Fig. 4) and higher spread as revealed from boxplots in (Fig. 5) in Zone-2. It vindicates the contributions from additional sources^2^ which is manifested as a greater spring density and greater (δ^18^O) in Zone-2 compared to Zone-1 and 3 and can also be accounted by a large mid altitude range of subzone **B** of Zone-2. However, unlike^2,5^ where the temporal permeability enhancement by the earthquakes caused the isotopic anomalies in spring water giving the indication of additional sources, the isotopic variation in the present work is a result of spatial permeability enhancement due to tectonically varied nature of thrusts^17^ in three Zones. The broad range of isotopic composition (more depleted to less depleted) in Zone-2 compared to other zones (Fig. 5), suggests different sources recharged by meteoric water at different elevations^2^. Possibly some of the sources with evaporation effects with less depleted isotopic compositions might reflect the dry season recharges^2^ and sources which are isotopically more depleted suggests the rainy season recharges^2^ thus validating the prevalence of different recharge zones providing the water to springs resulting high isotopic variations in Zone-2 than others (Fig. 5).

The present account also elucidates the geosystem services provided by thrust zones in delivering the water resources to the Himalayan community that is dependent on springs for their day-to-day water needs. The diversity in the study area with the presence and absence of the number of thrust lines culminates to identify the geosystem services associated with the thrust zones. It has been stressed by^20,21^ about the clear distinction between the biotic and abiotic services and has labelled the services provided by the geosystems as the geosystem services. They define the geosystem services as the “goods and services that contribute to human well-being specifically resulting from the subsurface”. Brilha et al. uses^39,40^ to delineate the geosystem services and has categorized it into regulating, supporting, provisioning, and cultural services under the ecosystem services while^20^ Gray et al. did the same classification under the abiotic ecosystem services which^18^ Gray has reclassified as Geosystem services into regulating, provisioning, supporting, cultural and knowledge^20,38^. further compartmentalizes the regulating services into global services (magnetic field or the gradual release of the Earth ‘s internal heat, hydrological cycle, and the carbon cycle) and local services like the river flows with input from groundwater to sustain the river flow in drought periods, weathering, erosion, river transport of sediments and nutrients, depuration of water during its underground circulation and many other terrestrial services^20^. The identified thrust zones associated with geosystem services in the present context can be added to the aforesaid criterion of^18,20,38^ under regulating services of regional and local scale as a contribution to the geosystem services classification and the crucial, inevitable, and underrated role of geosystem services to the society. Also, according to^43^ the spring water is the most important ecosystems that provides a multitude of goods and services to the human and biodiversity. Thus the springs provide irrigation water, potable water and livestock watering under provisioning services, cultural services in the form of medicinal services and treatment of diseases (digestive, skin, etc.) recreation (ecotourism) and supporting services in the form of stability of living beings including humans^43^.Albeit the springs being small in terms of absolute volumes of fresh water they have been a vital source of water for drinking and sanitation^44^ and hence their low amounts accounts for the higher dependence of communities in the Indian Himalayan Region^45^ and hence needs more attention for conservation. Thus, the springs as an important fresh water commodity in Himalayan region and their anomalous presence in the area controlled by tectonics, provides valuable insights for the need of targeted policy interventions in order to conserve them for accessing their long term benefits for the society.

## Conclusions

We found the greater spread of − 4‰ in oxygen isotopic compositions of Zone-2 and a lower spread of − 2‰ and − 2.8‰ in Zone-1 and Zone-3 respectively. The greater spread may be attributed to greater spring density and “highly tectonically active nature” of Zone-2. Greater spring density in Zone-2 is likely due to the permeability enhancement by the “highly tectonically active nature” of Zone-2^17^ than Zone-1 and 3 and has been designated as geosystem services of thrust zones. The study suggests the higher variation of stable isotopes of oxygen within the thrust zone (Zone-2) as an indicator of more water recharge into the zone which is manifested as dominance of very high spring density stretches in Zone-2 than others. Thus, the presence of the thrust zones in the study area functions to serve as abiotic assets to the Himalayan community by increasing spring density. These abiotic goods and services are the natural capitals for the Himalayan populace and are referred to as the geosystem services^18–23^, and their understanding is imperative to get multiple benefits for society in terms of water availability. Thus, we can say that Zone-2 has a higher density of springs and hence benefits most from the geosystem services of thrust dominance. The concept of oxygen isotope variation and its diversity within thrust zones has been introduced and linked to spring density and tectonically more or less active nature of Zones. The higher variation shows higher spread which in turn indicates additional recharge and different recharge sources in the vicinity of tectonically active zones compared to tectonically less active zones from water of springs. Albeit, the fruition of geosystem services as the springs in Zone-2 are a prerogative to the local community of area even then it’s further evaluation will be a pragmatic approach during the event of lean or drought periods. If geosystem services could continue to provide water even in low amounts that they are containing through precipitation, their role would become more crucial in sustaining the populace of the region. Nevertheless, from the present data and assessments it can be inferred that the permeability enhancements in the thrust zones can be targeted as suitable rainwater harvesting sites in order of the need to maintain environmental flows due to the immense services of springs for the Himalayan populace.Such soft engineering practice could benefit, so that apart from the natural regulations, sustainable human interventions can provide viable solutions for the growing water needs in the Himalayan terrains.

## Supplementary Information

Supplementary Information.
