# Supplementary material for: Relationship of isotopic variations with spring density in the structurally controlled springs and related geosystem services in Alaknanda Valley, Garhwal Himalaya, India
Source: Sci Rep. 2022 May 10;12:7679. doi: 10.1038/s41598-022-11762-z (PMC9091205; doi:10.1038/s41598-022-11762-z)
Supplement: Supplementary file 1 — Supplementary Information. [file 41598_2022_11762_MOESM1_ESM.pdf]

Supplementary Information File:

Title: Relationship of isotopic variations with spring density in the structurally controlled springs and related geosystem services in Alaknanda Valley, Garhwal Himalaya, India

Author list: Aakash Mohan Rawat[1*], Dhirendra Singh Bagri[1], Sudhir Kumar[2], Ruchi Badola[3], Syed Ainul Hussain[3]

[1]**Hemvati Nandan Bahuguna Garhwal University, Uttarakhand, India**

[2]**National Institute of Hydrology, Roorkee, Uttarakhand, India**

[3]**Wildlife Institute of India, Dehradun, India**

[*]**Corresponding author:** aakashrawat4444@gmail.com

Sampling locations_Table S1.

S.No.	Sample	Location	Altitude (in feets)
1	SP1	SANKRISHEERA	2663
2	SP2	JAKNET	2706
3	SP3	SUBASHNAGAR	2700
4	SP4	SUBASHNAGAR	2596
5	SP9	KARNPRAYAG	2484
6	SP10	KARNPRAYAG	2582
7	SP11	CHATWAPEEPAL	2554
8	SP12	CHATWAPEEPAL	2855
9	SP13	DHABA	2755
10	SP14	DHABA	2755
11	SP15	GALNAU	2630
12	SP16	GALNAU(CHTPPL)	2635
13	SP17	JHIRKOTI	2636
14	SP18	CHATWAPEEPAL	2460
15	SP19	PANNESHWAR MH	2481
16	SP20	ANGODHA	2529
17	SP21	ANGODHA	2529
18	SP22	CHAWKI KMEDA	2596
19	SP23	CHAWKI KMEDA	2775
20	SP24	KMEDA	2715
21	SP25	GHOLTIR	2275
22	SP26	GHOLTIR	2602
23	SP27	GHOLTIR	2602
24	SP28	GHOLTIR	2410
25	SP29	SHIVNANDI	2530
26	SP30	SHIVNANDI	2530
27	SP31	RATURA	2309
28	SP32	RATURA	1949

29	SP33	TILANI	2419
30	SP34	LAMEDI	2312
31	SP35	RUDRAPRAYAG	2319
32	SP36	GULABRAI	2319
33	SP38	BELNI	2078
34	SP39	RUDRAPRAYAG	2102
35	SP42	RUDRAPRAYAG	1989
36	SP43	RUDRAPRAYAG	1989
37	SP44	RUDRAPRAYAG	2044
38	SP49	RPG BYPASS	2333
39	SP50	RPG BYPASS	2333
40	SP51	RPG BYPASS	2313
54	SP75	BAJPUR	3400
55	SP76	BAJPUR	3396
56	SP77	BAJPUR	3165
57	SP78	KHUED	3221
58	SP79	MAITHANA	3384
59	SP80	MAITHANA	3174
60	SP81	PURSADI	3701
61	SP82	NANDPRAYAG	3698
62	SP83	NANDPRAYAG	3136
63	SP84	NANDPRAYAG	3136
64	SP85	NANDPRAYAG	3098
66	SP87	NANDPRAYAG	3057
67	SP88	SALNA	3037
68	SP89	NAULI	2950
69	SP90	NAULI	2978
70	SP91	NAULI	2978
71	SP92	VIRASKUNJ	2858
72	SP93	VIRASKUNJ	2858
73	SP94	KADESHWAR	2762
74	SP95	KADESHWAR	2784
75	SP96	KADESHWAR	2865
76	SP97	SIROLI	2797
77	SP98	SIROLI	2797
78	SP99	SIROLI	2797
41	SP52	NARKOTA	2234
42	SP53	KHANKRA	2382
43	SP54	KHANKRA	2390
44	SP55	FATAHPUR	2740
45	SP56	DUNGRIPANTH	2229
46	SP57	DUNGRIPANTH	2229

47	SP58	MARODA	2260
48	SP59	CHAMDHAR	2210
49	SP60	CHAMDHAR	2210
50	SP61	SWEET	1890
51	SP72	CHUNGI SRNGR	1777
52	SP73	KOTHAR DHARA	1945
53	SP74	SRINAGAR,Hanuman Mandir	1820
79	SP102	LAMBAGARH	7098
80	SP103	LAMBAGARH	6938
81	SP104	VINYCHATTI	6847
82	SP105	VENAKULI	6552
83	SP106	BALDHODA	5001
84	SP114	MARWADI	4872
85	SP115	MARWADI	5191
86	SP116	JHARKULA	5821
87	SP117	JHARKULA	5821
88	SP118	VRIDHBADRI	4982
89	SP119	ANIMATH	4781
90	SP120	HAILANG	4788
91	SP121	HAILANG	4788
92	SP122	GULABKOTI	4598
93	SP123	PATALGANGA	4682
94	SP124	GUNDALA	4532
95	SP125	GADORA	4031
97	SP126	MAYAPUR	3894
98	SP127	STONE CRUSHER	3262
